# Supplementary figures and images for: Misrouting of glucagon and stathmin-2 towards lysosomal system of α-cells in glucagon hypersecretion of diabetes
Source: Islets. 2021 Dec 20;14(1):40–57. doi: 10.1080/19382014.2021.2011550 (PMC8726656; doi:10.1080/19382014.2021.2011550)

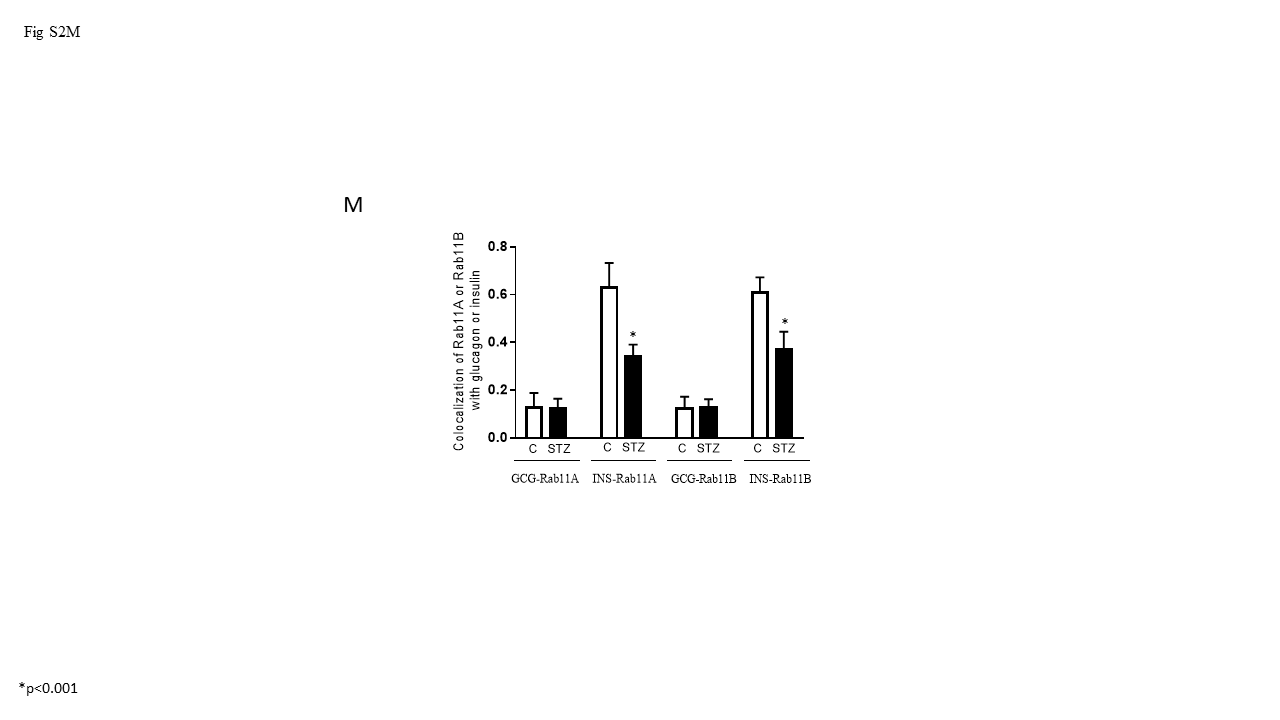

Supplement: Supplemental Material [file KISL_A_2011550_SM1730.zip › Fig. S2M.TIF]

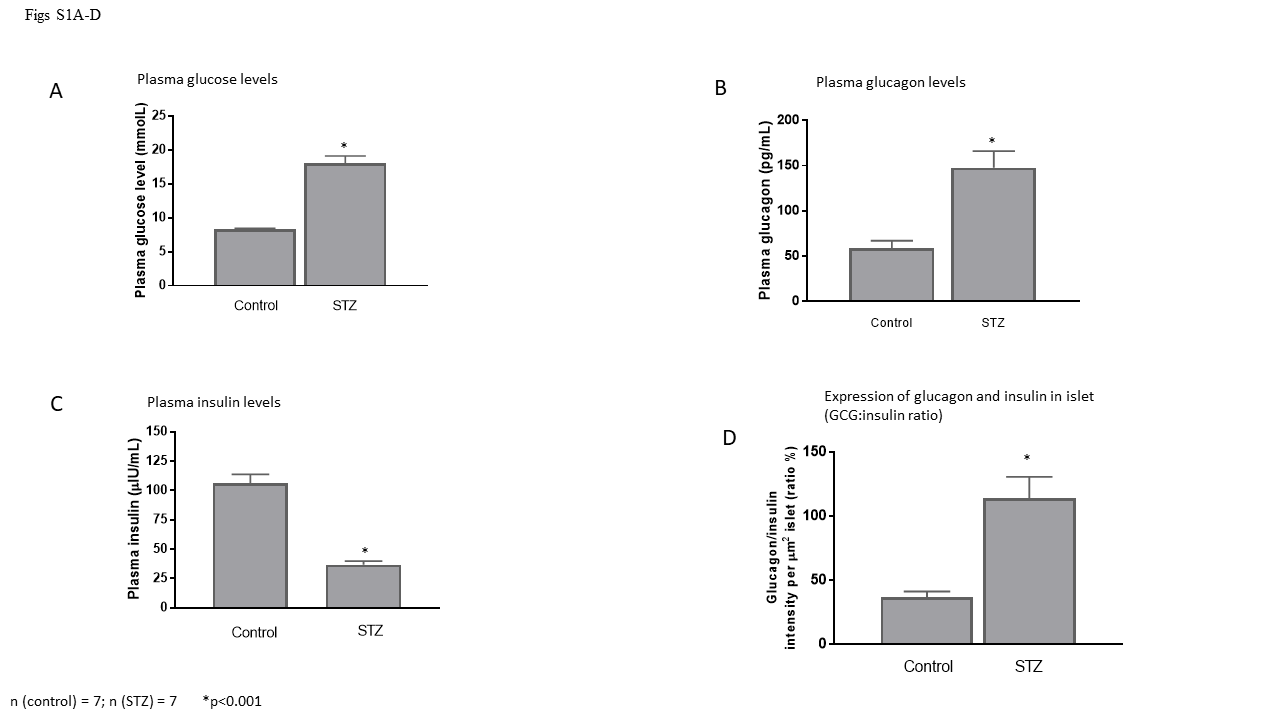

Supplement: Supplemental Material [file KISL_A_2011550_SM1730.zip › Figs S1A-D.TIF]

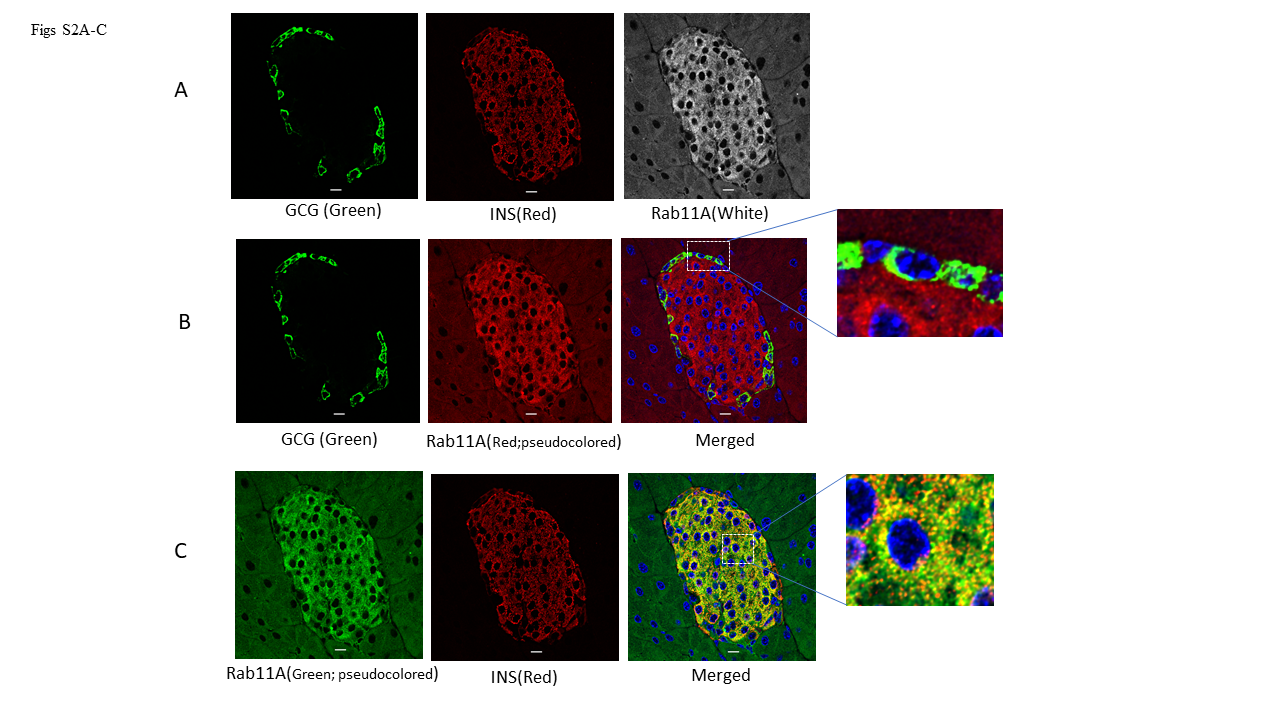

Supplement: Supplemental Material [file KISL_A_2011550_SM1730.zip › Figs S2A-C.TIF]

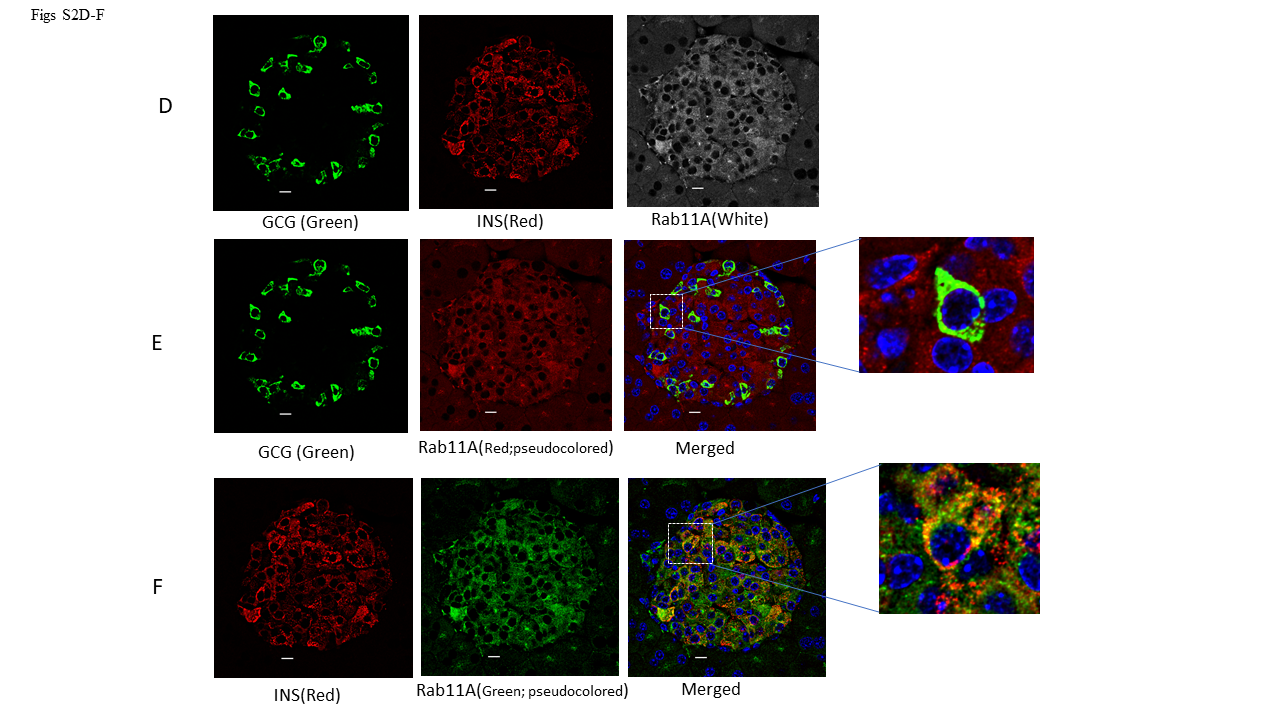

Supplement: Supplemental Material [file KISL_A_2011550_SM1730.zip › Figs S2D-F.TIF]

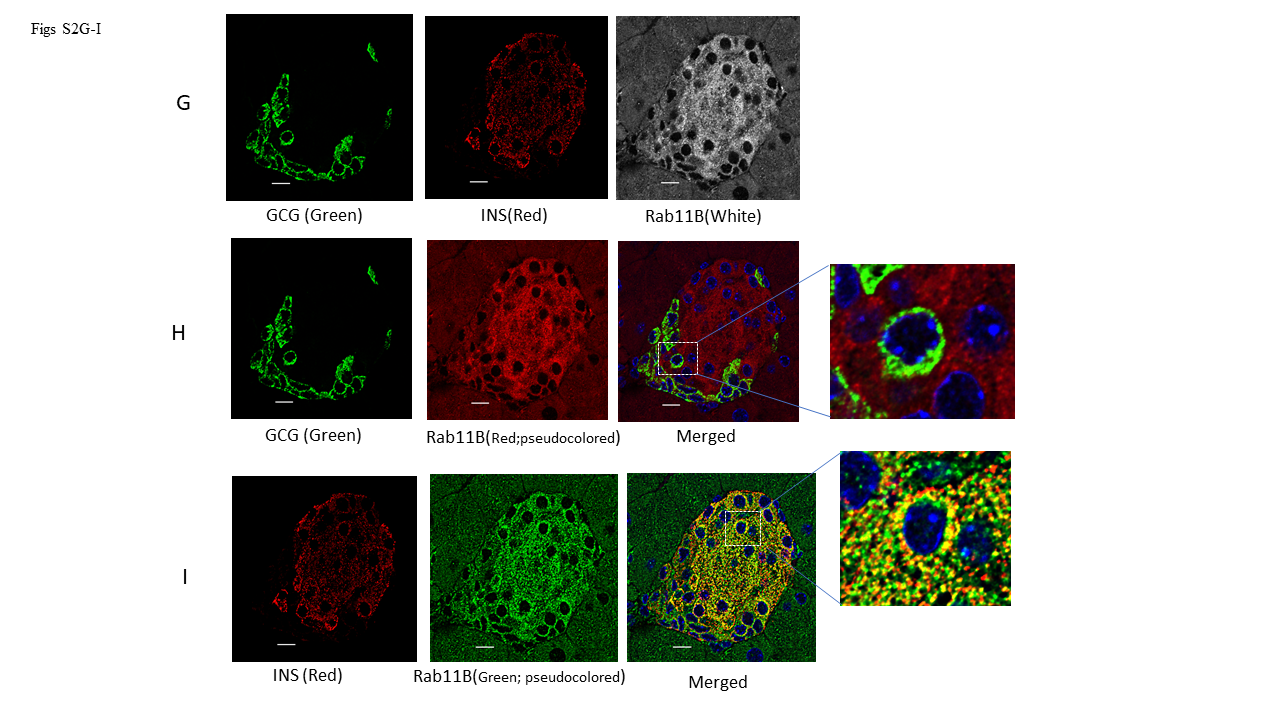

Supplement: Supplemental Material [file KISL_A_2011550_SM1730.zip › Figs S2G-I.TIF]

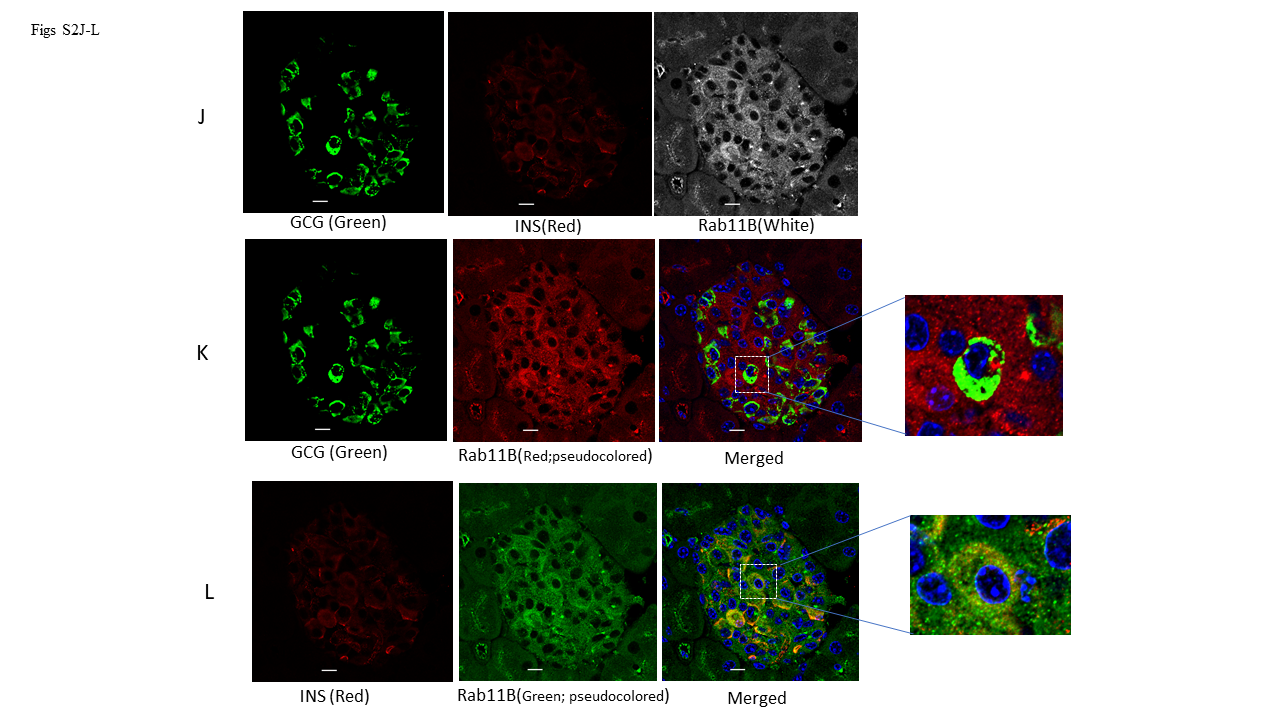

Supplement: Supplemental Material [file KISL_A_2011550_SM1730.zip › Figs S2J-L.TIF]
